# Calcium pyrophosphate crystals in L4‐L5 facet joint from small fluid sample

**DOI:** 10.1002/jgf2.520

**Published:** 2022-01-23

**Authors:** Yohei Kanzawa, Jun Ohnishi, Naoto Ishimaru, Saori Kinami

**Affiliations:** ^1^ 73897 Department of General Internal Medicine Akashi Medical Center Hyogo Japan

**Keywords:** calcium pyrophosphate deposition disease, facet

## Abstract

The image shows calcium pyrophosphate deposition disease‐induced facet joint arthritis in a Japanese female. In this case, an extremely small fluid specimen meant it was not possible for a full analysis set, but gram stain and culture allowed correct diagnosis. The patient was successfully treated with celecoxib.

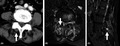

A 75‐year‐old woman presented with 2‐day history of right‐side lower back pain affecting movement and walking, which gradually progressed within 2 days. Her body temperature was 38.1°C, blood pressure 160/128 mmHg, heart rate 100 beats per minute, respiratory rate 30 breaths per minute, and oxygen saturation 94% while breathing ambient air. Tenderness was noted the on right side of the L3‐L5 spinous processes without swelling, but there was no tenderness of the spine. Enhanced computed tomography and MRI showed a low‐density area at the right L4‐L5 facet joint and inflammation in erector spinae muscles (Figure 1A‐C). Needle aspiration under fluoroscopy yielded only a tiny amount of yellowish opaque fluid, too little to order a full analysis set of joint fluid. Gram stain and bacterial culture were ordered to rule out infection. After collection of joint fluid and blood culture, ceftriaxone and vancomycin were initiated to treat infection. According to gram stain findings (Figure 2), celecoxib was administered to treat calcium pyrophosphate deposition (CPPD) facet arthritis with inflammation of erector spinae muscles. Blood and joint fluid cultures were negative, so antibiotics were discontinued. After treatment with celecoxib (100 mg twice a day), the patient's pain subsided, and she regained the ability to walk with a corset.

**FIGURE 1:**
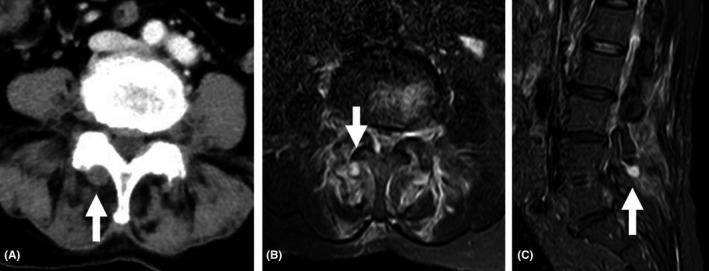
(A) Enhanced computed tomography shows a low‐density area in the right L4‐L5 facet joint (arrow). (B,C) MRI images show a high‐intensity area in the same region

**FIGURE 2:**
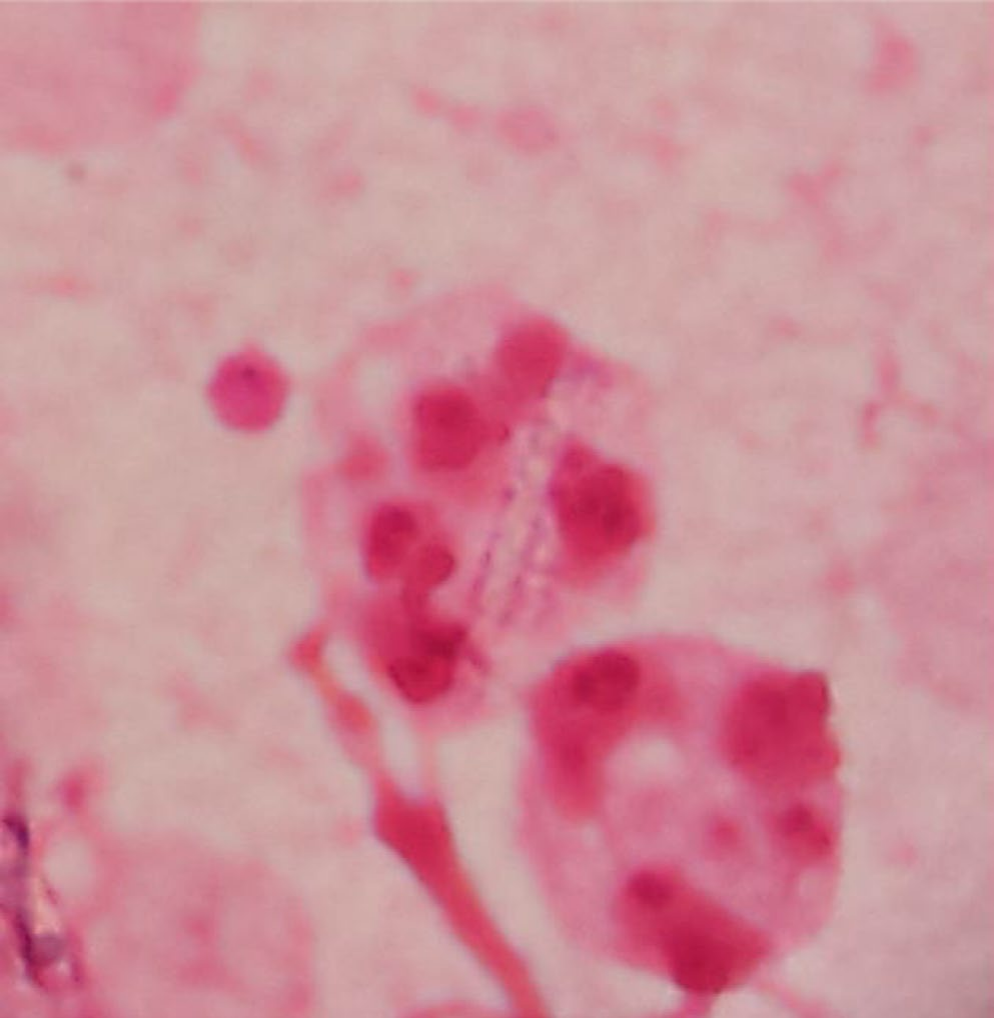
Gram stain shows calcium pyrophosphate crystals phagocyted by neutrophil without bacteria

An acute form of monoarticular arthritis is a common clinical presentation of CPPD,^1^ but other, less common clinical presentations of CPPD have also been described. Involvement of the spine in CPPD is uncommon, but can be sometimes seen in patients with extensive peripheral CPPD arthritis.^2^, ^3^ CPPD crystal deposits rarely extend into facet joints; isolated or multiple CPPD crystal deposits have been shown, but not always with acute arthritic symptoms.^4^, ^5^ CPPD involving the facet joint is rarely reported as a presentation of pseudo‐gout attack.^5^ In our case, the chief complaint was lower back pain because of attack of facet joint CPPD without peripheral lesion.

Inflammation of the facet joint is induced by various etiologies, such as infection, rheumatic disease (rheumatoid arthritis or seronegative spondyloarthropathy), and CPPD with crystal formation. Ruling out infection before diagnosis of CPPD is most important. CPPD deposits, which are typically shown as punctate, linear, or lumpy calcifications on CT, sometimes help in CPPD diagnosis, but aspiration and biopsy are the key to ruling out infection.^2^ In this patient, the fluid collection was of very low volume, but still enough to make a final diagnosis.

Our patient had a facet joint CPPD attack, suggested to be a differential diagnosis of low back pain. Aspiration to rule out infection was key to diagnosis and successful treatment.

## CONFLICT OF INTERESTS

The authors have stated explicitly that there are no conflicts of interest in connection with this article.

## INFORMED CONSENT

Written informed consent was obtained from the patient for publication of this case report.
